# Operative Versus Nonoperative Management of Appendicitis: A Long-Term Cost Effectiveness Analysis

**DOI:** 10.1177/2381468319866448

**Published:** 2019-08-17

**Authors:** Lindsay A. Sceats, Seul Ku, Alanna Coughran, Britainy Barnes, Emily Grimm, Matthew Muffly, David A. Spain, Cindy Kin, Douglas K. Owens, Jeremy D. Goldhaber-Fiebert

**Affiliations:** Stanford-Surgery Policy Improvement Research and Education (S-SPIRE) Center, Department of Surgery, Stanford University, Stanford, California; School of Medicine, Stanford University, Stanford, California; School of Medicine, Stanford University, Stanford, California; School of Medicine, Stanford University, Stanford, California; Department of Management Science and Engineering, Stanford University, Stanford, California; Stanford University, Stanford, California; Stanford University Medical Center, Stanford, California; Section of Acute Care Surgery, Department of Surgery, Stanford University, Stanford, California; Stanford-Surgery Policy Improvement Research and Education (S-SPIRE) Center, Department of Surgery, Stanford University, Stanford, California; Center for Primary Care and Outcomes Research, School of Medicine, Stanford University, Stanford, California; VA Palo Alto Health Care System, Palo Alto, California; Center for Primary Care and Outcomes Research, School of Medicine, Stanford University, Stanford, California

**Keywords:** appendicitis, cost-effectiveness analysis, laparoscopic appendectomy, nonoperative management

## Abstract

**Background.** Recent clinical trials suggest that nonoperative management (NOM) of patients with acute, uncomplicated appendicitis is an acceptable alternative to surgery. However, limited data exist comparing the long-term cost-effectiveness of nonoperative treatment strategies. **Design.** We constructed a Markov model comparing the cost-effectiveness of three treatment strategies for uncomplicated appendicitis: 1) laparoscopic appendectomy, 2) inpatient NOM, and 3) outpatient NOM. The model assessed lifetime costs and outcomes from a third-party payer perspective. The preferred strategy was the one yielding the greatest utility without exceeding a $50,000 willingness-to-pay threshold. **Results.** Outpatient NOM cost $233,700 over a lifetime; laparoscopic appendectomy cost $2500 more while inpatient NOM cost $7300 more. Outpatient NOM generated 24.9270 quality-adjusted life-years (QALYs), while laparoscopic appendectomy and inpatient NOM yielded 0.0709 and 0.0005 additional QALYs, respectively. Laparoscopic appendectomy was cost-effective compared with outpatient NOM (incremental cost-effectiveness ratio $32,300 per QALY gained); inpatient NOM was dominated by laparoscopic appendectomy. In one-way sensitivity analyses, the preferred strategy changed when varying perioperative mortality, probability of appendiceal malignancy or recurrent appendicitis after NOM, probability of a complicated recurrence, and appendectomy cost. A two-way sensitivity analysis showed that the rates of NOM failure and appendicitis recurrence described in randomized trials exceeded the values required for NOM to be preferred. **Limitations.** There are limited NOM data to generate long-term model probabilities. Health state utilities were often drawn from single studies and may significantly influence model outcomes. **Conclusion.** Laparoscopic appendectomy is a cost-effective treatment for acute uncomplicated appendicitis over a lifetime time horizon. Inpatient NOM was never the preferred strategy in the scenarios considered here. These results emphasize the importance of considering long-term costs and outcomes when evaluating NOM.

Appendectomy has traditionally been the mainstay of treatment for uncomplicated appendicitis. While usually safe and effective, appendectomy is not totally benign: surgery incurs perioperative risk and has been associated with short- and long-term complications, with reported rates ranging from 2% to 23%.^1–4^ Given these risks, multiple randomized trials have assessed the efficacy of managing appendicitis nonoperatively using antibiotics alone.^5–12^

Proponents of nonoperative management (NOM) contend that it is less expensive than appendectomy, as it minimizes operative costs and may reduce hospitalization days. Trials have estimated the costs of NOM versus surgical management for the index hospitalization, but have not assessed long-term costs of recurrence or other complications.^5,9,13^ The necessary observation period to assess these long-term outcomes is impractical for prospective trials,^14^ and certainly exceeds individual surgeons’ typical episodes of care. However, accurate assessment of the cost-effectiveness of NOM warrants consideration of the long-term costs of appendicitis-related care, including the lifetime potential for recurrence, ongoing risk for appendiceal malignancy, or potential postoperative complications such as adhesive bowel obstructions. Two prior cost-effectiveness analyses compared NOM with appendectomy in the adult and pediatric populations,^15,16^ but these analyses were completed over 1-year time horizons and did not model long-term costs.

To determine the cost-effectiveness of NOM versus operative management of acute uncomplicated appendicitis, we conducted an analysis comparing two NOM strategies against laparoscopic appendectomy. A novel aspect of our analysis is the modeling of both short- and long-term costs over a reference patient’s lifetime to study the enduring effects of NOM and surgery. This analysis is intended to inform surgeons and third-party payers regarding long-term economic implications when comparing NOM and laparoscopic appendectomy as first-line therapies for uncomplicated appendicitis.

## Methods

This computer simulation modeling study was deemed exempt from human subject review because it uses only publicly available, deidentified, and aggregate data sources.

### Base Case

We first defined the reference case as a healthy 20-year-old male in the United States presenting to the emergency room with imaging-confirmed uncomplicated appendicitis. Uncomplicated appendicitis was defined as inflammation of the appendix in the absence of abscess, phlegmon, perforation, fecalith on imaging, or diffuse peritonitis on initial clinical exam.

### Model

**Figure 1:**
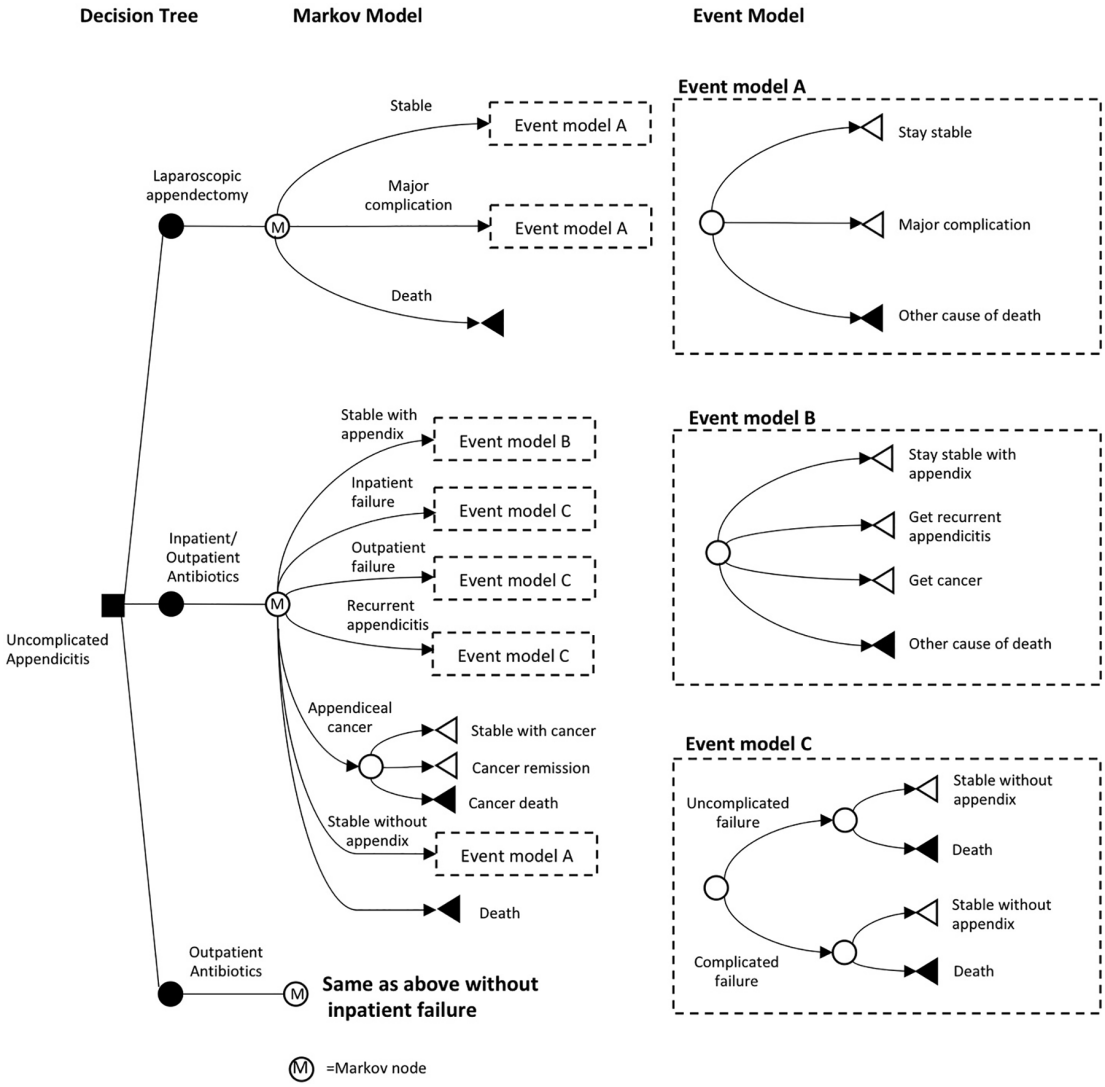
Simplified model schema for patients with uncomplicated appendicitis.

We constructed a decision model using decision analysis software (Treeage, Williamstown, MA). We used literature review and consultation with experts to build a decision tree comparing three management strategies: 1) laparoscopic appendectomy, 2) inpatient NOM with 3-day hospitalization for intravenous antibiotics (piperacillin-tazobactam) followed by completion course of 7 days of outpatient oral antibiotics (amoxicillin-clavulanate), and 3) outpatient NOM with 7 days of oral outpatient antibiotics only (amoxicillin-clavulanate) (Figure 1). For each strategy, outcomes were modeled based on probabilities derived from prior randomized trials and other published literature. Antibiotic regimens were based on those used in randomized trials.^5,11^ Two NOM arms were included as we wished to consider both the most typical nonoperative treatment strategy as well as the potentially least resource-invasive, as recent pilot studies have shown comparable outcomes between inpatient and outpatient management strategies.^17^ Appendectomy was considered the reference treatment, as it is the current standard of care.

Inpatient failure of NOM was defined as symptom progression during the index hospital admission. Outpatient failure was defined as symptom progression or return of symptoms after discharge from the index hospitalization within 30 days of initial presentation. Treatment failures occurring after 30 days were considered to be recurrent appendicitis. Complicated failure or recurrence was defined as perforation with associated abscess. We considered short-term perioperative complications including ileus, surgical site infection, anesthetic complications, and postoperative hemorrhage. We considered long-term complications including trocar-site hernia and adhesive small bowel obstruction. Finally, we accounted for the possibility that appendicitis may be the first presentation of an otherwise unrecognized appendiceal tumor,^4,18,19^ and NOM may result in a missed opportunity to treat an early, confined appendiceal tumor. Recent data suggest that appendiceal tumors are incidentally discovered in around 1% of appendectomies.^4,20–22^ It is thus reasonable to consider the potential downstream effects of leaving an unidentified tumor in situ following NOM over the rest of a patient’s life.

We also conducted additional scenario analyses to explore how much various factors would influence the assessment of NOM. These included the following: 1) if an older patient underwent NOM (if the base case was instead an average 40- or 65-year-old), 2) if patients who were managed nonoperatively never developed appendiceal cancer (i.e., if all appendiceal masses were seen on initial imaging and treated operatively), 3) if patients who were managed nonoperatively never failed or recurred, and 4) if all failures or recurrences were uncomplicated (i.e., could be managed by appendectomy alone).

The time horizon for the model was the expected lifetime of the reference case, derived from US life tables.^23^ Age-appropriate background mortality and costs were considered in the model.^24,25^ We used Markov modeling with a cycle length of 1 month to examine probabilities of long-term events over the expected lifespan of the patient. After initial chance nodes accounted for events and costs occurring in the first 30 days, Markov nodes incorporating half-cycle correction were used. Incremental cost-effectiveness ratios were compared with a $50,000 per QALY (quality-adjusted life-year) gained willingness-to-pay threshold.^26^ For the strategies considered, we examined the incremental cost-effectiveness ratios (ICERs)—defined as the incremental cost of a given strategy divided by its incremental benefit compared with the next best alternative. Future costs and life-years were discounted at an annual rate of 3%.^27^

### Probabilities

Probabilities were obtained from literature review (Table 1); these were preferentially drawn from randomized trials comparing NOM and appendectomy. Values not obtainable in these trials were derived from other published data, meta-analyses, the American College of Surgeons National Surgical Quality Improvement Program risk calculator,^28^ and expert opinion from clinicians. Base case values were felt by clinicians to represent the best available estimates from the literature.

**Table 1:** Model Parameters

Parameter	Baseline Value	Range	Distribution	References
*Probabilities*				
Short-term postoperative complication	0.072	0.008–0.198	Beta	7–11, 37, 48–51
Long-term postoperative complication (annual)	0.005	0.0014–0.008	Beta	48, 52–54
Perioperative mortality	0.0005	0.0005–0.004	Beta	6, 28, 37, 51
Mortality after long-term postoperative complication	0.066	0.023–0.141	Beta	55
Failure NOM on inpatient antibiotics	0.092	0.058–0.179	Beta	6, 7, 9, 10
Complicated failure NOM on inpatient antibiotics	0	n/a	Beta	6, 9
Outpatient failure NOM after inpatient antibiotics	0.142	0.055–0.228	Beta	6, 7, 9, 10
Complicated outpatient failure NOM after inpatient antibiotics	0.01	0–0.061	Beta	6,7,9
Failure NOM on outpatient oral antibiotics	0.138	0.067–0.21	Beta	5, 11
Complicated failure NOM on outpatient oral antibiotics	0.052	0.02–0.331	Beta	5
Recurrence after NOM	Year 1: 0.273 Year 2: 0.34 Year 3: 0.352 Year 4: 0.371 Year 5: 0.391 After Year 5: recurrence risk equal to appendicitis risk in the general population: 0.086	0.044–0.391	Beta	5–7, 12, 56
Complicated recurrence after NOM	0.268	0–0.312	Beta	7–11
Mortality during NOM	Background mortality	n/a	Beta	24
Mortality after appendectomy for complicated appendicitis	0.00599	0–0.01	Beta	57–59
Appendiceal malignancy after appendicitis presentation	0.008	0.0004–0.036	Beta	4, 6, 7, 20–22
Mortality from appendiceal adenocarcinoma	Year 1: 0.086 Year 2: 0.13 Year 3: 0.174 Year 4: 0.218 Year 5: 0.26	n/a	Log-normal	60
Remission from appendiceal adenocarcinoma	Year 1: 0.90 Year 2: 0.83 Year 3: 0.75 Year 4: 0.72 Year 5: 0.69	n/a	Log-normal	61
Mortality from appendiceal carcinoid	Year 1: 0.033 Year 2: 0.056 Year 3: 0.079 Year 4: 0.102 Year 5: 0.126	n/a	Log-normal	62
Remission from appendiceal carcinoid	Year 1: 0.95 Year 2: 0.92 Year 3: 0.85 Year 4: 0.75 Year 5: 0.70	n/a	Log-normal	63
*Costs (2017 US$)*				
Laparoscopic appendectomy	7606	3,803–11,409	Normal	29, 30
Inpatient NOM	7369	3684.5–11053.5	Normal	29, 30, 32
Outpatient NOM	169	84.5–253.5	Normal	32
Percutaneous drain	13,323	6661.5–19984.5	Normal	29, 30
Emergency room visit	923	461.5–1384.5	Normal	31
Short-term postoperative complication	8431	4215.5–12646.5	Normal	29, 30
Long-term postoperative complication	11,060	5,530–16,590	Normal	29, 30
Right colectomy for malignancy	14,362	7,181–21,542	Normal	29, 30
12 cycles FOLFOX chemotherapy for malignancy	38,276	19,138–57,414	Normal	29, 30, 32, 64
*Utilities (quality-adjusted life months)*				
Laparoscopic appendectomy	0.85	0.63–0.98	Beta	65
Laparoscopic appendectomy with complication	0.76	0.23–0.99	Beta	66
Postoperative outpatient recovery	0.85	0.63–0.98	Beta	65
Successful NOM	0.93	0.78–0.99	Beta	65
Failure NOM	0.81	0.76–0.85	Beta	65
Recurrent appendicitis	0.72	0.67–0.76	Beta	65
Interval appendectomy	0.74	0.71–0.77	Beta	65
Malignancy	0.78	0.21–0.99	Beta	67

NOM, nonoperative management.

### Costs

Costs were considered from the perspective of a third-party payer. Procedural costs were calculated using the 2017 Centers for Medicare and Medicaid Services (CMS) physician fee schedule.^29^ We summed the physician work, practice expense, and malpractice relative value units (RVUs) and multiplied by a conversion factor of 35.8887 (per CMS data) for the Current Procedural Terminology (CPT) codes relevant to our study (44970 = laparoscopic appendectomy; 49406 = percutaneous drainage of abdominal abscess; 49560 = hernia repair; 44143 = hemicolectomy; 10180 = postoperative wound infection). Hospitalization costs were obtained from the CMS final rule for fiscal year 2017,^30^ and were calculated by multiplying the conversion factor by the sum of the RVUs for 1 admit day (CPT 99222), 1 discharge day (CPT 99239), and any intervening hospital days (CPT 99231). Average length of hospitalization per procedure was obtained from literature review. We multiplied the sum of the labor ($3420), non-labor ($2096), and capital cost rates ($447) by the appropriate DRG (diagnosis-related group) multiplier. The costs of emergency department evaluation and follow-up clinic visits were obtained from the CMS Hospital Outpatient Prospective Payment System.^31^ The sum of hospital charges and physician charges for a level 4 visit (CPT 99284) was added to the cost of imaging; assuming that half of the patients received an abdominal computed tomography scan and half received an ultrasound. Medication costs were obtained from the Federal Supply Schedule.^32^ The average wholesale price per medication was multiplied by 0.64 to obtain the net final price paid by Medicaid for each medication (per Congressional Budget Office). The Medicaid final price includes distribution and dispensing costs. Costs used in our analysis are summarized in Table 1.

### Utilities

Utilities related to hospitalizations, surgical or procedural interventions, and recovery from appendicitis or appendectomy were drawn from published literature as available. Previously published studies assessing age-related background decline in health-related quality of life was used to estimate the baseline utility of the reference case.^33^

### Assumptions

We assumed that all appendectomies were completed laparoscopically, in line with modern case series describing very low rates of conversion to open appendectomy for uncomplicated appendicitis.^34–37^ We assumed that all NOM failures or recurrences underwent laparoscopic appendectomy (i.e., no further attempts at NOM were made). All patients with complicated failures or recurrences were assumed to undergo percutaneous drain placement by interventional radiology followed by interval appendectomy. We assumed that 70% of patients returning with an appendiceal malignancy presented with a carcinoid tumor >2 cm and underwent right hemicolectomy, and the remaining 30% presented with Stage III appendiceal adenocarcinoma and underwent right hemicolectomy followed by 12 cycles of FOLFOX chemotherapy.^18,38–40^ We purposefully assumed that patients would present with advanced-stage malignancies due to a delay in cancer diagnosis related to NOM. As a small percentage of patients undergoing appendectomy may be found to have malignancies at that time, we assumed that these patients would be cured by appendectomy alone and would not require further treatment. We felt this assumption was reasonable, as we defined our reference case as having confirmed appendicitis without concerning imaging findings correlated with diagnosis of later-stage malignancy during appendectomy.

### Sensitivity Analyses

We first conducted one-way deterministic sensitivity analyses to evaluate how the decision might change across the plausible range of each model parameter given its uncertainty while holding all others at their base case values. We used sensitivity analyses to assess the importance in changes of utilities for health states individually and in combination, while preserving the preference ordering of health states. Baseline values were varied over the ranges displayed in Table 1. Probabilities were varied over the broadest range obtainable from the literature or the 99th percentile confidence interval calculated using the baseline value (whichever was wider). Costs were varied from 50% to 150% of the base case cost. Individual utilities were varied across the 95th percentile confidence intervals of beta distributions formed for their means. Two-way sensitivity analysis was performed to examine the combined effect of varying rates of short-term NOM failure (<30 days) and late recurrence of appendicitis after NOM (within 5 years).

We then conducted Monte Carlo probabilistic sensitivity analyses using beta distributions for input probabilities and utility weights and normal distributions for defined procedural costs. Univariate utility distributions were then correlated to establish joint uncertainty distributions exploiting ordinal preferences over health states, in order to avoid bias and improve probabilistic sensitivity analyses.^41^ The uncertainty for normal distributions was calculated using a previously defined method.^42^ Values were repeatedly sampled from their appropriate uncertainty distributions 10,000 times.

Authors’ funding sources had no role in the study.

## Results

### Base Case

When considered over the expected lifetime of the base case patient, outpatient NOM proved the least costly and generated the fewest QALYs and life-years (Table 2). Both laparoscopic appendectomy and inpatient NOM were more expensive but generated more QALYs and life-years compared with outpatient NOM. Taken together, laparoscopic appendectomy proved cost-effective compared with outpatient NOM at the willingness-to-pay threshold considered (ICER $32,300 per QALY gained), while inpatient NOM was dominated by laparoscopic appendectomy.

**Table 2:** Total Costs, QALYs, and ICERs for Base Case and Scenario Analyses

Scenario	Management Strategy	Cost (US$)		Life Years		QALY		ICER (US$ per QALY)
		Total	Incremental	Total	Incremental	Total	Incremental	
Base case: Age 20	Laparoscopic appendectomy	236,200	2500	26.5976	0.0709	25.0043	0.0773	32,300
	Inpatient NOM	241,000	7300	26.5272	0.0005	24.9279	0.0009	Dominated
	Outpatient NOM	233,700	NA	26.5267	NA	24.9270	NA	NA
Base case: Age 40	Laparoscopic appendectomy	301,200	2800	22.1180	0.0577	20.4625	0.0638	43,900
	Inpatient NOM	305,800	7400	22.0608	0.0005	20.3995	0.0008	Dominated
	Outpatient NOM	298,400	NA	22.0603	NA	20.3987	NA	NA
Base case: Age 65	Laparoscopic appendectomy	320,800	3300	13.1668	0.0315	11.7035	0.0380	86,800
	Inpatient NOM	325,300	7800	13.1356	0.0003	11.6661	0.0006	Dominated
	Outpatient NOM	317,500	NA	13.1353	NA	11.6655	NA	NA
No cancer risk after NOM	Laparoscopic appendectomy	236,200	2200	26.5976	−0.0022	25.0043	0.0030	733,300
	Inpatient NOM	241,400	7400	26.6003	0.0005	25.0021	0.0008	Dominated
	Outpatient NOM	234,000	NA	26.5998	NA	25.0013	NA	NA
No risk of NOM failure or recurrence	Laparoscopic appendectomy	236,200	7500	26.5976	0.0438	25.0043	0.0435	172,400
	Inpatient NOM	235,800	7100	26.5538	0	24.9608	0	Dominated
	Outpatient NOM	228,700	NA	26.5538	NA	24.9608	NA	NA
No risk of complicated NOM failure or recurrence	Laparoscopic appendectomy	236,200	6400	26.5976	0.0479	25.0043	0.0480	133,300
	Inpatient NOM	237,700	7900	26.5469	−0.0028	24.9532	−0.0031	Dominated
	Outpatient NOM	229,800	NA	26.5497	NA	24.9563	NA	NA

ICER, incremental cost-effectiveness ratio; NA, not applicable (referent group); NOM, nonoperative management; QALY, quality-adjusted life-years.

### Scenario Analyses

We first assessed whether the cost-effectiveness of NOM was sensitive to the age of the reference patient considered. When the age of the reference case was increased to either age 40 or age 65, laparoscopic appendectomy remained the most effective strategy and was cost-effective at age 40 but not at age 65 (ICERs $43,900 and $86,800 per QALY gained, respectively).

When we considered the situation where the risk of developing appendiceal cancer after NOM was zero (i.e., if it was assumed that all appendiceal masses would be captured on initial imaging and thus managed operatively), appendectomy’s health benefits relative to NOM gained by removing the appendix were attenuated (Table 2). Hence, outpatient NOM remained the least costly, and both inpatient and outpatient NOM increased the life-years delivered. Appendectomy was no longer cost-effective (ICER $733,300), and inpatient NOM was dominated by laparoscopic appendectomy.

When we considered the scenario where all NOM was successful (i.e., the risk for failure or recurrence of NOM was zero), the costs of both NOM strategies decreased compared with the base case (Table 2). Outpatient NOM remained the least costly. The life expectancy and QALYs associated with both NOM strategies increased, although appendectomy continued to generate the greatest life-years and QALYs. Inpatient NOM cost more than outpatient NOM without generating greater efficacy, while laparoscopic appendectomy was not cost-effective (ICER $172,600 per QALY).

Finally, we considered the situation where all failures and recurrences were uncomplicated and proceeded directly to appendectomy instead of requiring initial percutaneous drainage followed by interval appendectomy. The cost of both NOM strategies decreased relative to the base case and outpatient NOM remained the least costly. QALYs and life-years increased for both NOM strategies; appendectomy continued to generate more life-years and QALYs. In this scenario, laparoscopic appendectomy was no longer cost-effective compared with outpatient NOM (ICER $133,300 per QALY). Inpatient NOM was dominated by laparoscopic appendectomy.

### Sensitivity Analyses

Model results were sensitive to changes in the following parameters in one-way sensitivity analyses: perioperative mortality rates during appendectomy, the probability of developing recurrent appendicitis and the probability that the recurrence be complicated, the probability of developing an appendiceal malignancy after NOM, and the cost of laparoscopic appendectomy. When the probability of perioperative mortality during laparoscopic appendectomy was >0.26%, outpatient NOM dominated laparoscopic appendectomy. When the probability of developing recurrent appendicitis in the 5 years following NOM was >26%, laparoscopic appendectomy dominated. Laparoscopic appendectomy also dominated when the probability of a complicated appendicitis recurrence was >4.6%. Laparoscopic appendectomy dominated outpatient NOM when the long-term risk of developing an appendiceal malignancy after NOM was <0.48%. Varying the cost of laparoscopic appendectomy also changed the preferred strategy: when greater than $10,173 (baseline $7,606), outpatient NOM was preferred over laparoscopic appendectomy. In no sensitivity analysis that we considered was inpatient NOM the cost-effective strategy.

**Figure 2:**
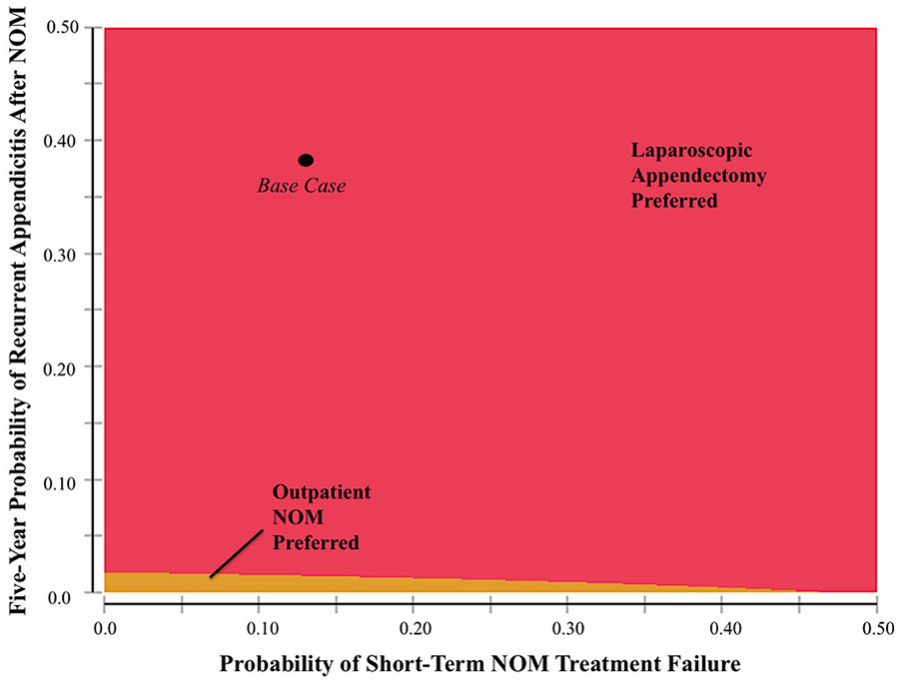
Two-way sensitivity analysis showing the preferred management strategy when varying the probability of short-term, nonoperative management (NOM) failure and appendicitis recurrence.

We conducted a two-way sensitivity analysis when the probabilities of short-term NOM failure and long-term appendicitis recurrence were varied concurrently. Figure 2 shows situations where outpatient NOM would be preferred to laparoscopic appendectomy, namely, when recurrence and failure rates are low. None of the points where outpatient NOM was preferred were inside the 95% confidence intervals of the failure and recurrence rates from recent randomized trials. Inpatient NOM was never the preferred treatment strategy in the two-way sensitivity analyses considered here.

**Figure 3:**
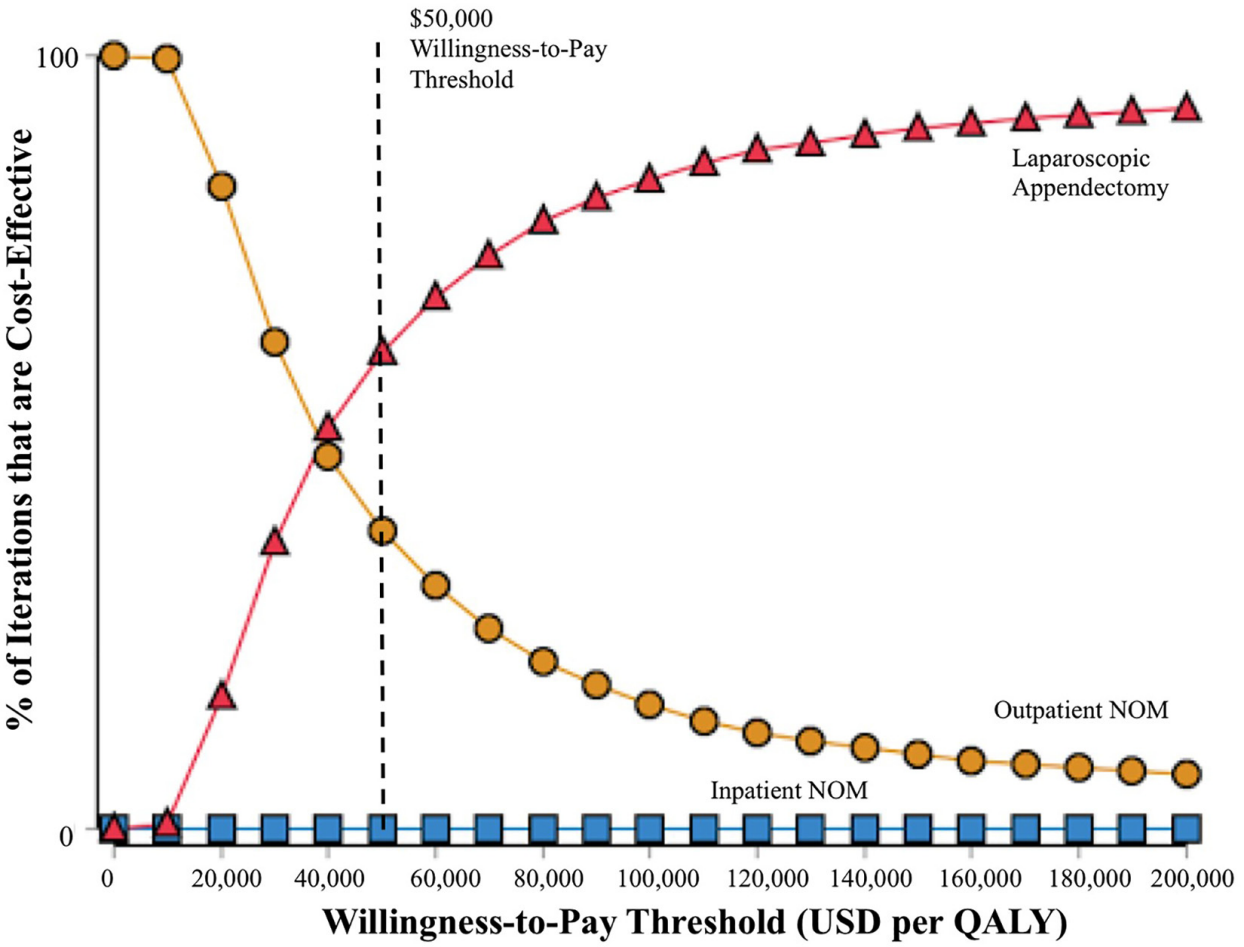
Cost-effectiveness acceptability curves from probabilistic sensitivity analysis.

In probabilistic sensitivity analyses, laparoscopic appendectomy was the preferred strategy in 62% of 10,000 simulated cases when the willingness-to-pay threshold was set at $50,000 per QALY, while outpatient NOM was preferred in the other 38%. A cost-effectiveness acceptability curve is included as Figure 3.

## Discussion

Our analysis finds that laparoscopic appendectomy is the most effective management strategy for acute uncomplicated appendicitis when the lifetime effects of appendicitis-related treatment are considered. It is cost-effective compared with outpatient NOM with an ICER of $32,300 per QALY. Inpatient NOM is the costliest strategy and was less effective than laparoscopic appendectomy.

These findings are important as few other studies have modeled or assessed the long-term health and economic consequences of NOM for uncomplicated appendicitis. Prior cost-effectiveness analyses assessing NOM were limited to a 1-year time horizon and suggested that NOM dominated laparoscopic appendectomy.^15,16^ A cost analysis of the APPAC trial comparing appendectomy to NOM concluded that NOM incurred fewer costs over the duration of the trial.^13^ In contrast, our model suggests that laparoscopic appendectomy is a cost-effective treatment option despite the additional costs associated with surgery when the lifetime implications of appendicitis-related treatment are considered. Conversely, inpatient NOM as conventionally defined by prospective trials was not cost-effective over a lifetime horizon. This demonstrates the importance of collecting long-term data from registries and prospective trials assessing NOM. Prospective trials of short duration and individual surgeons’ personal experiences will not account for long-term outcomes and may make NOM appear overly favorable.

When considered over a patient’s lifetime, the differences in costs and QALYs between appendectomy and NOM were relatively small. This likely reflects the overall excellent outcomes following appendicitis and low rates of long-term complications following either management strategy. However, the sensitivity and scenario analyses presented here are informative in determining important factors in the long-term cost-effectiveness of appendicitis treatment. Appendectomy cost and perioperative mortality proved influential in one-way sensitivity analyses, suggesting that minimizing operative costs and mortality are important targets in real-life practice. Model outcomes were insensitive to changes in age until age 65, suggesting that these results are applicable to a broad adult population.

The probabilities of developing and dying from appendiceal malignancies were strongly influential on treatment preferences in this analysis, despite their relatively rarity. Appendectomy was preferred over outpatient NOM when the probability of finding an incidental appendiceal malignancy at index appendicitis presentation was greater than 0.59%. Recent data suggest a 1.1% probability of incidentally discovering an appendiceal malignancy at index appendicitis presentation for patients aged 18 to 65, and a 2.7% probability for ages >65.^4^ Although rare, it is thus important to consider the possibility of future malignancy when considering NOM.^6,7,20–22^

As expected, rates of NOM treatment failure and appendicitis recurrence were influential on model outcomes and the preferred strategy. Two-way sensitivity analyses revealed that the threshold rates of treatment failure and appendicitis recurrence resulting in dominance of outpatient NOM over laparoscopic appendectomy were at the low end of recurrence rates seen in randomized trials, where treatment failure rates have reached as high as 38%.^5–12^ The thresholds of NOM failure and appendicitis recurrence resulting in dominance by laparoscopic appendectomy are notably lower than those reported in prior cost-effectiveness analyses conducted over a 1-year time horizon, which found that NOM was not the preferred strategy when short- and long-term failure rates ranged from 32% to 45%.^15,16^ These results suggest that treatment failure rates of large-scale pending prospective trials should be analyzed closely as they bear significant financial implications for the cost-effectiveness of NOM, and that careful tracking of long-term recurrence rates is important.

Notably, inpatient NOM as defined in this analysis was never cost-effective or the preferred strategy compared with outpatient NOM or laparoscopic appendectomy. The definition of inpatient NOM was modeled to be consistent with the largest randomized trial to date: a 3-day admission for intravenous antibiotics followed by 7 days of completion oral antibiotics.^6^ Although not yet common clinical practice, recent pilot studies have moved toward assessing fully outpatient treatment strategies, consistent with the least resource-invasive strategy presented here.^17^ Clinicians must be aware that NOM protocols that result in hospitalizations longer than hospitalizations typical for laparoscopic appendectomies (i.e., 24 hours or longer) are unlikely to prove cost-effective on a population level.

We recognize the limitations associated with this study, particularly the relatively limited prospective data used to generate model parameters. This is especially true for parameters involving long-term probabilities, including the long-term risk of appendicitis recurrence after NOM and long-term risks of postoperative complications. Every effort was made to preferentially use model parameters drawn from high-quality randomized data, only supplementing with retrospective data and expert clinician opinion if no other parameter estimates were available. Health state utilities used as model parameters were limited in availability and drawn from single studies, and thus may significantly influence model outcomes. Health care costs in the United States are known to exceed costs in other developed nations^43,44^; thus, cost estimates and their effects on the model may not be generalizable to other settings. Parameters used to model outpatient NOM were drawn from prospective trials that included an all-oral antibiotic regimen, as no major prospective trials to date have tested a fully outpatient NOM strategy. For the purposes of studying the costs associated with outpatient treatment, we assumed that oral antibiotics given in hospital would yield similar results to oral antibiotics taken at home. However, this may be unrealistic as patients may derive additional benefit from hospitalization alone, making outpatient management appear overly effective in this analysis.

Finally, the model is built on the assumption that patients have imaging-confirmed appendicitis, consistent with modern surgical practice. Rates of negative appendectomy have decreased significantly secondary to the high diagnostic accuracy of modern diagnostic imaging.^35,45–47^ These results may not be applicable to low-resource settings where preoperative diagnostic imaging is less frequently utilized and rates of negative appendectomy are higher.

## Conclusions

In this cost-effectiveness analysis of treatment strategies for acute uncomplicated appendicitis, laparoscopic appendectomy is the most effective management strategy for acute uncomplicated appendicitis when considered over a lifetime horizon and is cost-effective compared with outpatient NOM. In cases where there is a desire not to perform surgery, outpatient NOM should be considered, as inpatient NOM as previously defined in randomized trials is costlier and is not cost-effective.
